# The ACA training programme to improve communication between general practitioners and their palliative care patients: development and applicability

**DOI:** 10.1186/1472-684X-11-9

**Published:** 2012-06-27

**Authors:** Willemjan Slort, Annette H Blankenstein, Bernardina S Wanrooij, Henriëtte E van der Horst, Luc Deliens

**Affiliations:** 1 Department of General Practice, EMGO + Institute for Health and Care Research, VU University Medical Center, Amsterdam, The Netherlands; 2 Department of General Practice/Family Medicine, Division of Public Health, Academic Medical Center, University of Amsterdam, Amsterdam, The Netherlands; 3 Department of Public and Occupational Health, EMGO + Institute for Health and Care Research, VU University Medical Center, Amsterdam, The Netherlands; 4 End-of-Life Care Research Group, Ghent University & Vrije Universiteit Brussel, Brussels, Belgium

**Keywords:** Palliative care, Communication, Education, Family practice, Feasibility studies, Physician-patient relations

## Abstract

We describe the development of a new training programme on GP-patient communication in palliative care, and the applicability to GPs and GP Trainees. This ‘ACA training programme’ focuses on ** *A* ***vailability* of the GP for the patient, ** *C* ***urrent issues* that should be raised by the GP, and ** *A* ***nticipating* various scenarios. Evaluation results indicate the ACA training programme to be applicable to GPs and GP Trainees. The ACA checklist was appreciated by GPs as useful both in practice and as a learning tool, whereas GP Trainees mainly appreciated the list for use in practice.

## What this paper contributes to our knowledge about education of providers regarding palliative care

Previous studies identified factors reported by palliative care patients, their relatives, GPs or end-of-life consultants as relevant for GP-patient communication in palliative care. In this study we summarized these factors into a 19-items ACA checklist, divided into three categories: [1] the *availability* of the GP for the patient, [2]*current issues* that should be raised by the GP, and [3] the GP *anticipating* various scenarios. Moreover, we evaluated the newly developed ACA training programme and found that this training programme appears to be applicable to practising GPs and inexperienced GP Trainees. The ACA checklist was appreciated by GPs as useful both in practice and as a learning tool, whereas GP Trainees mainly appreciated the list for use in practice. Future research should assess the effectiveness of the training programme.

## Training programme on GP-patient communication in palliative care

Although there are differences between countries, general practitioners (GPs) often play a central role in providing palliative care. Palliative care refers to the total care that is provided for a patient and his/her family when the patient has a life-threatening disease that no longer responds to curative treatment. GPs involved in palliative care need to be skilful in communicating with patients, their families, and care-givers. Communicating with palliative care patients has been acknowledged to be more difficult than communicating with patients with less serious conditions, [1] because communication in palliative care involves a complex mix of medical, psychosocial and spiritual issues within the context of impending death. Physicians, including GPs, often fail to communicate effectively with patients about palliative care issues, [2,3] and most GPs have never received any training in communication skills with a specific focus on palliative care at all throughout their career [4,5]. Moreover, there is still no evidence-based training programme available to improve the skills of GPs and GP Trainees (GPTs) in their communication with palliative care patients.

In the Palliative Care Centre of Expertise at the VU University Medical Center we designed a new training programme for GP-patient communication in palliative care. The results of our recent studies yielded three categories of factors reported to be relevant for GP-patient communication in palliative care: the *availability* of the GP for the patient, *current issues* that should be raised by the GP, and the GP *anticipating* various scenarios [6,7]. We used the first letters of the three categories (ACA) as an acronym for the training programme.

The first objective of this paper is to describe the development of this ‘ACA training programme’ to improve GP-patient communication in palliative care. The second objective is to evaluate the first experiences of a group of GPs and a group of GPTs with this new training programme, in order to formulate recommendations for its future use.

## Development of the ACA training programme

We designed a new training programme for GP-patient communication in palliative care, including the following educational components deduced from two recent reviews: the programme is learner-centred, using several methods, carried out over a longer period of time, mostly in small groups to encourage more intensive participation, combining theoretical information with practical rehearsal and constructive feedback from peers and skilled facilitators [8,9].

To support this new training programme we developed a checklist, based on the results of a systematic review [6] and qualitative study [7] which we have conducted previously to identify factors reported by palliative care patients, their relatives, GPs or end-of-life consultants as relevant for GP-patient communication in palliative care.

Table 1 shows the original article(s) from which it was derived for each item of the ACA checklist. In our qualitative study most of the factors identified in the review were confirmed, but as indicated in Table 1 the items ‘paying attention to physical symptoms’, ‘wishes for the present and the coming days’, ‘unfinished business’, and ‘offering follow-up appointments’ were additional to the results of the review. From all identified factors we selected the facilitating aspects of the communicative behaviour of a GP providing palliative care and the issues that should be raised by the GP, and we summarized these factors into the 19 items of the ACA checklist. We divided these items into three categories: [1] the *availability* of the GP for the patient, [2]*current issues* that should be raised by the GP, and [3] the GP *anticipating* various scenarios (ACA).

**Table 1:** **The ACA checklist (*A* *vailability-* *C* *urrent issues-* *A* *nticipating* ), factors derived from our recent systematic review**[6]**and/or qualitative study**[7]

**ACA checklist**	**From review [source]**	**From qualitative study**[7]
***A* *vailability* ***(of the GP for the patient):*		
1. taking time	X [10-16]	X
2. allowing any subject to be discussed	X [2,14,15,17,18]	X
3. active listening	X [14-17,19-21]	X
4. facilitating behaviour (*e.g.* empathic, respectful, attentive, occasionally also phoning or visiting the patient spontaneously)	X [2,10-17,19-23]	X
5. shared decision-making with regard to diagnosis and treatment plan	X [13,17,20,24,25]	X
6. accessibility (*e.g.* phone numbers)	X [11,13,14,23]	X
***C* *urrent issues* ***(that should be raised by the GP):*		
7. diagnosis	X [10,13,15,17,20,24-28]	X
8. prognosis	X [10,13,15-17,20,24-28]	X
9. patient’s complaints and worries:- physical	-	X
10. - psychosocial	X [13,18,25,28]	X
11. - spiritual	X [22,28,29]	X
12. wishes for the present and the coming days	-	X
13. unfinished business, bringing life to a close	-	X
14. discussing treatment and care options (concerning 7–13)	X [13,17,19,24,25,28]	X
***A* *nticipating* ***(various scenarios):*		
15. offering follow-up appointments	-	X
16. possible complications	X [28]	-
17. wishes for the coming weeks/months (personal wishes as well as preferences with regard to medical decisions)	X [17,19,21,28]	X
18. the actual process of dying (final hours/days)	X [11,14,18,21,22,25]	-
19. end-of-life decisions	X [14,19,21,28]	X

The GP should apply all six items concerning availability during each visit, because these items can be considered as necessary conditions for effective communication. The eight items for ‘current issues’ and the five items for ‘anticipating’ should be explicitly addressed by the GP, but not necessarily all during one visit. It seems even preferable to spread discussion about these 13 issues over several visits, allowing GP and patient to take the necessary time for each issue. During every visit the GP and the patient can identify and discuss those issues on the ACA checklist which are most relevant for the patient at that moment. GPs can use the ACA checklist in practice in the following ways: [1] using the checklist before and during a palliative care consultation gives an overview of the issues that can be addressed; [2] after a series of consultations the checklist can be used to check if all essential issues are discussed with the patient; [3] GPs or consultants can use the checklist to detect possible causes of problems in communication.

The ACA training programme was established to enable GPs and GPTs to:

· obtain knowledge about ACA communication skills

· achieve better insight into (individual shortcomings in) their communication skills

· improve their ACA communication skills

· develop self-education skills, using the ACA checklist as a tool for self-assessment of their communication skills.

For the eight steps of the ACA training programme, see Table 2.

**Table 2:** The consecutive steps of the ACA training programme (and the estimated time spent by participants on each step)

**At the start of the ACA training programme; at the residential course:**	
**Step 1**	Each participating GP or GP Trainee (GPT) had a *videotaped physician-patient interview* with a trained actor simulating a patient in an advanced stage of lung or colon cancer, according to a detailed script; immediately after the interview the participant received general feedback on communication style from the actor (30 minutes).
**Step 2**	*Instructions on the ACA checklist,* using oral presentations and written information (ACA booklet) in order to enhance the understanding of the participants of effective GP-patient communication in palliative care; each participant also received a plastic chart of the ACA checklist for use in daily practice (30 minutes).
**Within two months after the start of the programma, outside the residential course:**	
**Step 3**	All participants received *feedback according to the ACA checklist* on their performance during the videotaped physician-patient interview in step 1. The GPs received individual written feedback from an experienced facilitator, the GPTs received oral feedback from their peers and facilitators in small groups (60 minutes).
**Between the start of the programme and halfway through the programme, outside the residential course:**	
**Step 4**	The participants were asked to enhance their understanding of the ACA checklist and their insight into their own communication skills by *studying* the written information, *discussing* this material with their peers in small groups, and *trying out* newly acquired skills in their own general practice to identify problem areas from their own experience (60 minutes).
**Before the residential course at halfway through the programme; outside the residential course:**	
**Step 5**	The participants were asked *to formulate learning goals* based on the individual shortcomings in their ACA communication skills identified at all previous steps (30 minutes).
**Halfway through the programme; at the residential course:**	
**Step 6**	All participants were offered *role-play exercises* tailored to their individual learning goals. Hence, they could practise the desired behaviour in the safe environment of small groups and with the help of feedback on their performance from their peers and facilitators. GPs performed role-play with actors simulating a patient, GPTs performed role-play with other participants in the course, which had the additional advantage of enabling them to experience the position and emotions of the patient (60 minutes).
**At the end of the ACA training programme; at the residential course:**	
**Step 7**	Each participant had a *second videotaped interview* with an actor simulating a patient; immediately after the interview the participant again received general feedback on communication style from the actor (30 minutes).
**Step 8**	All participants could use the second videotaped interview and the ACA checklist as tools for *self-assessment* of their communication skills, and they could then (off course) formulate new learning goals and start a new learning cycle (60 minutes).
The estimated total duration of all steps in the ACA training programme is six hours.	

## Applicability of the ACA training programme

### Two settings

We evaluated the applicability of the ACA training programme in two groups with different characteristics: practising GPs who attended a 2-year Palliative Care Peer Group Training Course, and inexperienced GPTs from two vocational training institutes.

The training programme for the GPs took place during the first year of a two-year Palliative Care Peer Group Training Course. This course consisted of four two-day residential courses, followed by two-hour peer group sessions with five GPs in each group, facilitated by a palliative care consultant, every six to eight weeks. The GPs who enrolled for this study were participants in two such courses affiliated with the Comprehensive Cancer Centres of Eindhoven and Rotterdam, which started in 2006 and 2007, respectively. Most of the steps in the ACA training programme were conducted by the regular facilitators of the course, supervised by one of the authors (BW); steps 2 and 3 of the programme were conducted by the first author (WS).

The training programme for the GPTs took place during the first six months of the third year of their vocational training. In this final year the trainees worked for 3–4 days a week in the practice of their vocational GP trainer, and on one day a week they attended training programmes at their vocational training institute. Each group consists of approximately 10 trainees, facilitated by a GP and a behavioural scientist. The GPTs who enrolled for this study were participants in five such groups that started between October 2007 and March 2008 (two groups at the VU University Medical Center in Amsterdam and three groups at the University Medical Centre in Utrecht). The ACA training programme was, as recommended by Reinders *et al.*, [30] conducted by the regular teachers in the vocational GP training institutes, who had received detailed instructions about the training programme from the first author (WS).

### Time schedule of the ACA training programme

Steps 1 and 2 (see Table 2) were planned on the first day of the training programme. Within two months after the first day all participants received individual feedback on their videotaped simulation interview (= step 3). During the following months they had to complete step 4 in order to formulate their personal learning goals (= step 5). Six months after the start of the programme, the GPs participated in role-play exercises which were tailored to their learning goals (= step 6); the GPTs performed their role-play exercises 3–4 months after the start of their programme. Finally, a second interview with an actor simulating a patient was videotaped, so that the participants could subsequently use this to assess their communication skills against the ACA checklist.

### Characteristics of the participants

The following data on the participating GPs were recorded at baseline: gender, age, years of experience in general practice, group, duo, or single-handed practice, urban or rural practice, working part-time or full-time, vocational GP trainership, courses on palliative care attended during the previous two years, and number of palliative care patients in the GP practice who had died during the previous year at any location.

The following data on the participating GPTs were recorded at baseline: gender, age, group, duo or single-handed vocational practice, urban or rural vocational practice, part-time or full-time vocational training, specific experience in palliative care, and number of palliative care patients for whom the GPT had provided palliative care during the first year of vocational training.

### Attendance and appreciation of the ACA training programme

At the end of the ACA training programme all participating GPs and GPTs were asked to complete an evaluation form. To assess the applicability of the programme we evaluated the rate of attendance of GPs and GPTs and their appreciation of the different steps of the programme. Steps 7 and 8 were not included in this evaluation, because the forms were completed directly before step 7. At first, we developed an evaluation form for the GPs to score their appreciation on a 10-point Likert scale ranging from one (= no appreciation at all) to 10 (= maximal appreciation). Afterwards, this form was adapted for the GPTs to the format of evaluation forms that were customary at the vocational training; therefore, GPTs scored on a 5-point Likert scale ranging from one to five. For presenting the results in the outcome table, the scores of the GPs were divided by two to equalize these scores to those of the GPTs. For each step of the programme the scores were reported as mean scores (and standard deviations) for GPs and GPTs separately. We also asked the participants to indicate their learning goals and the aspects of the programme which facilitated or inhibited the learning process to their experience.

## Findings

### Characteristics of the participants

Of the 62 participating GPs, 45% were female, their mean age was 48, they had an average of 17 years of experience as a GP, and 64% were working in a (semi-)rural area. Of the 50 GPTs who completed the questionnaire at baseline, 72% were female, their mean age was 31, and 48% were working in a (semi-)rural area. Other characteristics are presented in Table 3.

**Table 3:** Socio-demographic and professional characteristics of participating general practitioners (GPs) and general practitioner trainees (GPTs)

**Characteristics of participants**	**GPs, N = 62**	**GPTs, N = 50**^**1**^
Gender, female N (%)	28 (45%)	36 (72%)
Age, mean (range)	48 (33-60)	31 (26–47)
Years of experience as a GP, mean (range)	17 (1–34)	n.a.^2^
Group or single-handed (vocational) practice		
- group practice, N (%)	24 (39%)	16 (32%)
- duo practice, N (%)	23 (37%)	20 (40%)
- single-handed practice, (%)	15 (24%)	14 (28%)
(Vocational) practice location area urban or rural		
- urban, N (%)	22 (36%)	26 (52%)
Working or attending vocational training part-time or full-time^3^		
- part-time, N (%)	32(52%)	11 (22%)
Vocational GP trainers, N (%)	17 (27%)	n.a.
Courses in palliative care attended by GP during the previous two years, N (%)	31(50%)	n.a.
Specific experience of GPT in palliative care at baseline, N (%)	n.a.	16 (32%)
GP estimate of number of palliative care patients in the practice who died during the previous year, mean (range)^4^	8 (1–40)	n.a.
GPT estimate of number of palliative care patients for whom GPT provided palliative care during the first year of vocational training, mean (range)	n.a.	2 (0–5)

^1^ four GPTs did not complete their form (holiday 2x and unknown reason 2x); ^2^ n.a. = not applicable; ^3^ full-time = 90-100%; ^4^ one GP answered ‘don’t know’.

### Response to the evaluation form

The GP response to the evaluation form was 85% (= 53/62). Nine participants in the course did not respond for the following reasons: one had become ill, one form was filled in but got lost, two GPs did not complete the form because they considered that certain components of the ACA training programme had disrupted other parts of the Palliative Care Peer Group Course, and five did not respond for unknown reasons, despite several requests.

The GPT response to the evaluation form was 67% (= 36/54). Reasons for non-response were absence at the final session (pregnancy leave 5x, illness 3x, holiday 2x, other course on the same day 2x, and unknown reason 2x), and 4 GPTs (from one group) did not complete the form because they had missed several steps of the programme.

### Attendance and appreciation of the ACA training programme

Steps 1-3a and 6 were attended by 87-100% of the GPs. Although 94% of the GPs studied the written feedback according to the ACA checklist, only 57% watched the video-recording of their interview. A smaller percentage of GPs (55-79%) completed the various parts of step 4, which they were asked to do ‘at home’, outside the residential courses. The various steps of the training programme were attended by 78-94% of the GPTs.

We estimated that each participant required six hours to complete all steps of the programme (see Table 2).

GPs appreciated all steps with mean scores ranging from 3.5 to 3.9 on a 1–5 scale. The mean GPT scores ranged from 2.9 to 4.0. For all steps the GP scores were higher than the GPT scores. The responding GPs and GPTs appreciated most the videotaped interview with feedback (steps 1 and 3), the role-play to practise individual learning goals (step 6), and the use of the ACA checklist in practice (step 4c). Among GPTs we found rather low appreciation scores for the use of the ACA checklist as a learning tool (studying the ACA booklet, formulation of individual learning goals, and applying the ACA checklist in discussions with vocational GP trainer or peers). For attendance and appreciation of all steps of the ACA training programme, see Table 4.

**Table 4:** Attendance and appreciation of the ACA training programme by responding general practitioners (GPs, N = 53) and general practitioner trainees (GPTs, N = 36)¹

**Steps of the ACA training programme**	**GPs attendance**	**GPs appreciation scores 1-5**^**2**^**, mean (SD)**	**GPTs attendance**	**GPTs appreciation scores 1-5**^**2**^**, mean (SD)**
Step 1a: Videotaped interview	100%	3.8 (0.5)	92%	3.7 (0.6)
Step 1b: Oral feedback from actor	100%	3.9 (0.5)	92%	3.5 (0.8)
Step 2a: Oral presentation on ACA checklist (GPs only)	98%	3.5 (0.6)	n.a.^3^	n.a.
Step 2b: Usefulness of content of ACA booklet *(GPTs only)*	n.a.	n.a.	94%	3.9 (0.7)
Step 3a: Written feedback on videotaped interview *(GPs only)*	94%	3.6 (0.5)	n.a.	n.a.
Step 3b: DVD of the videotaped interview *(GPs only)*	57%	3.7 (0.4)	n.a.	n.a.
Step 3c: Interactive feedback on videotaped interview *(GPTs only)*	n.a.	n.a.	81%	4.0 (0.4)
Step 4a: Studying the ACA booklet	79%	3.8 (0.4)	83%	2.9 (0.9)
Step 4b: Applying the ACA checklist in peer group discussions	55%	3.6 (0.4)	92%	3.0 (0.9)
Step 4c: Using the ACA checklist in palliative practice	68%	3.7 (0.4)	89%	3.6 (0.9)
Step 4d: Applying the ACA checklist in discussions with vocational GP trainer *(GPTs only)*	n.a.	n.a.	89%	3.2 (1.0)
Step 5: Formulation of individual learning goals *(GPTs only)*	n.a.	n.a.	83%	2.9 (1.2)
Step 6: Role-play to practise individual learning goals	87%	3.9 (0.5)	78%	3.6 (0.9)
Overall satisfaction with ACA training programme *(GPTs only)*	n.a.	n.a.	94%	3.5 (0.8)

^1^ Response was 85% for GPs and 67% for GPTs; ^2^ Scores from one (= no appreciation at all) to 5 (= maximal appreciation); ^3^ n.a. = not applicable.

The five most frequently spontaneously reported GP learning goals (8x or more) were: active listening, allowing any subject to be discussed, anticipating, wishes for the coming weeks/months, and using the ACA checklist as a guide. The GPTs most frequently reported using the ACA checklist as a guide (12x) and active listening (6x).

The two facilitating factors of the programme that GPs most frequently reported spontaneously were the peer group sessions (13x) and the ACA checklist (12x). The interview with an actor, the feedback, and seeing many palliative care patients in practice during the course were mentioned four times. The facilitating factor most frequently reported by the GPTs was the interactive feedback (according to the ACA checklist) on the video-taped interview (5x).

The inhibiting factors most frequently spontaneously reported by the GPs were only very few palliative care patients in their practice during the course (11x) and not enough time available for the training programme (10x). Inhibiting factors reported by the GPTs were that medical elements were lacking in the programme (5x) and that not all steps in the programme had been addressed (3x). During the 6 months duration of the programme the GPTs provided palliative care for an average of two patients (range 0–5).

## Discussion

### Main findings

We developed the ACA training programme to improve communication between GPs and their palliative care patients, consisting of eight consecutive steps, and based on three key areas of attention in communication: ** *a* ***vailability* of the GP for the patient, ** *c* ***urrent issues* that should be raised by the GP, and ** *a* ***nticipating* various scenarios. The results of this study show that the programme appears to be applicable to practising GPs who attended a 2-year Palliative Care Peer Group Training Course and to (inexperienced) GPTs from five vocational training groups. The ACA checklist was appreciated by GPs as useful both in practice and as a learning tool, whereas GPTs mainly appreciated the list for use in practice. A quarter of the GPs and a third of the GPTs spontaneously reported the ACA checklist to be a useful guide for communication with palliative care patients.

### Strengths and limitations of this study

Both content and educational approach of the ACA training programme are evidence-based. The content of the ACA training programme is based on the results of recent studies among palliative care patients, their relatives, GPs, and end-of-life consultants. The educational approach was derived from two systematic reviews of methods in training programmes for communication in palliative and cancer care.

Attendance and appreciation of the training programme were evaluated for each step of the programme.

The newly developed training programme was assessed among practising GPs and inexperienced GPTs. The GPs participated in a two-year Palliative Care Peer Group Training Course, and probably had a more than average commitment to palliative care, unlike the GPTs, who participated as part of their vocational training, with no special commitment. This might explain the moderate GPT response rate (67%) and their lower scores for appreciation. The appreciation scores of the two groups can only be compared with caution, because the GPs scored their appreciation on a 10-point scale and the GPTs on a 5-point scale. Non-responding GP(T)s might have had lower attendance rates and lower appreciation scores.

Although we evaluated the applicability of the ACA training programme in two different settings, our results can only be generalised with caution to use of the programme in other settings.

This study was a merely quantitative evaluation of the training programme; a qualitative study might have given additional insight in factors that would facilitate or inhibit effectiveness of this training programme.

The applicability was assessed with evaluation forms that were completed at the end of the training programme; registration of attendance and appreciation during the course might have yielded more accurate data.

### Comparison with existing literature

In their review of educational interventions in palliative care for primary care physicians, Alvarez *et al.* state that key elements of GP-patient communication in palliative care should be designed more specifically to obtain favourable results, and that effective training methods in key communication skills for doctors should be addressed in three phases: cognitive input, modelling, and practising key skills with feedback about performance [8]. These statements are in line with our findings that the GPs and GPTs appreciated the checklist with the 19 items and also the diverse methods in the ACA training programme.

Acquiring new consultation skills requires time. Blankenstein *et al.* found that GPs needed 20 hours of training and feedback sessions to learn how to apply new consultation skills aimed at somatising patients [31]. In our study, 10 GPs reported that they did not have enough time available for the ACA training programme. The estimated total duration of six hours for the programme might be too short.

### Recommendations for trainers

This study revealed possibilities to improve the applicability of the ACA training programme. Because the GPTs appreciated using the ACA checklist in practice more than using it as a learning tool, we recommend that first they try out the checklist in practice or role-play and afterwards reflect on their experiences with peers or their GP trainer. Therefore, the GP trainers should receive detailed instructions about the training programme like the regular teachers in the vocational GP training institutes. Because the attendance of the GPs to discussions about the ACA communication skills in their peer group was low, the facilitators of the peer groups should receive more training. As suggested by several GPTs, we recommend that the ACA training programme should be combined with training programmes for other medical and palliative care issues such as the Palliative Care Peer Group Training Course for GPs. Providing care for many palliative care patients in daily practice during the training period probably enhances the learning process for GP(T)s.

We were surprised that even a well-known communication skill such as ‘active listening’ was chosen by several experienced GPs as their main individual learning goal. We consider the opportunities for GP(T)s to assess their individual shortcomings in communication skills and to participate in role-play exercises tailored to their own learning goals as strong characteristics of the ACA training programme. The use of a checklist to clarify individual learning goals to facilitate the learning process might be extended to other topics and educational areas.

## Conclusions

The ACA training programme appears to be applicable to GPs and GPTs. Future research should assess the effectiveness of the ACA training programme with regard to GP(T) behaviour as well as patient outcomes.

## Competing interests

The funding bodies had no involvement in or influence on the study, and there are no conflicts of interests to be declared.

## Ethics committee

The study protocol was approved by the Medical Ethics Committee of the VU University Medical Center.

## Funding body

The GP study was funded by the Comprehensive Cancer Centres of Amsterdam and Eindhoven, CZ Healthcare Insurances, Pfizer bv, and the Janivo Foundation. The GPT study was funded by the Dutch Foundation for the Vocational Training of General Practitioners.

## Pre-publication history

The pre-publication history for this paper can be accessed here:

http://www.biomedcentral.com/1472-684X/11/9/prepub
